# An extensive skin defect and necrotizing fasciitis derived from a neglected epidermal cyst on the upper back: A case report

**DOI:** 10.1097/MD.0000000000049664

**Published:** 2026-07-17

**Authors:** Jae Hyun Kim, Jun Il Kim, Chan Min Chung

**Affiliations:** a Department of Plastic and Reconstructive Surgery, Hallym Sacred Heart Hospital, Hallym University College of Medicine, Anyang-Si, Korea.

**Keywords:** epidermal cyst/ fasciitis, necrotizing/ wound healing/

## Abstract

**Rationale:**

Epidermal cysts are one of the most prevalent skin lesions encountered in clinical practice. Although generally harmless, complications may arise when epidermal cysts are neglected, leading to potentially severe consequences, such as necrotizing fasciitis. Here, we report an unusual case of an extensive soft-tissue defect and necrotizing fasciitis resulting from an untreated epidermal cyst on the upper back.

**Patient Concerns:**

A 37-year-old male patient presented with a 12 × 10 cm epidermal cyst on his back with severe inflammation. The patient was also diagnosed with diabetes mellitus, which raised the need for comprehensive medical and surgical evaluations for treatment.

**Diagnoses:**

Magnetic resonance imaging revealed a broad soft-tissue defect with necrotizing fasciitis involving the back muscles.

**Interventions:**

Extensive debridement was performed, and various approaches were used to address the challenging wound size. After debridement, a soft-tissue defect measuring 35 × 30 cm was identified, and negative-pressure wound therapy followed by split-thickness skin grafting was used to cover the wound.

**Outcomes:**

At the 6-week post-surgery follow-up at the outpatient clinic, the patient exhibited a completely healed wound, and the patient’s uncontrolled diabetes mellitus was managed.

**Lessons:**

This case underscores the importance of not overlooking epidermal cysts and considering surgical intervention when they result in substantial skin and soft-tissue defects.

## 1. Introduction

Epidermal cysts, also known as epidermoid cysts, are common benign lesions originating from the epidermis. These cysts often result from the proliferation of epidermal cells within the dermis, forming a sac filled with keratin and sebum. The exact prevalence rates vary, although a slight male predominance is typically observed. Epidermal cysts most commonly occur on the face, neck, scalp, trunk, and less commonly on the scrotum, genitalia, and extremities. They are known to occur across all age groups, with a higher frequency noted in adulthood.^[1]^ Pathophysiologically, these cysts arise from occlusion of the follicular infundibulum or traumatic implantation of epidermal cells into the dermis. The development of epidermal cysts is often associated with factors such as trauma, genetic predisposition, and hormonal influences.^[2]^

Imaging modalities, such as ultrasound and magnetic resonance imaging, may be used to confirm the diagnosis and evaluate the extent of involvement, especially when complications are suspected.^[3]^ Epidermal cysts can occasionally lead to inflammation or rupture, showing tenderness, erythema, and drainage of a foul-smelling, cheesy substance. Although they rarely result in widespread skin and soft-tissue defects, neglected epidermal cysts can lead to severe, life-threatening conditions, such as necrotizing fasciitis.^[4,5]^

This report describes a rare case of a patient with extensive soft-tissue infection and necrotizing fasciitis originating from a neglected epidermal cyst on the upper back, which necessitated further surgical intervention. The patient’s clinical course involved a sequence of interventions, including surgical debridement, application of negative-pressure wound therapy (NPWT), and ultimately successful wound coverage achieved through reconstruction with a split-thickness skin graft (STSG). This multifaceted approach to wound management demonstrates the complexity of treating rare but advanced soft-tissue infections.

## 2. Case report

The purpose of this case report is to describe a rare and severe complication of a neglected epidermal cyst leading to necrotizing fasciitis on the upper back. We aim to highlight the diagnosis, clinical course, and surgical management of this condition.

A 37-year-old male patient presented to the emergency department with a large epidermal cyst on his upper back. The patient reported having the back mass for the past 10 days, accompanied by pain and general weakness. Initially diagnosed with 2 epidermal cysts, each measuring approximately 2 × 3 cm, at a local dermatology clinic, the patient received topical treatment and antibiotics for 7 days. However, the patient perceived the condition as trivial and chose not to take any medication, leaving the epidermal cyst untreated. Consequently, the symptoms worsened, and an expanding inflammatory process led the patient to visit our emergency department. Upon primary examination, the epidermal cyst showed signs of severe inflammation, with pus discharge and a foul odor. The cyst measured approximately 12 × 10 cm, with an opening of approximately 5 mm at the center. The surrounding area exhibited a heating sensation, and the patient had a fever with a body temperature of 38 °C. Laboratory results at admission revealed a white blood cell (WBC) count of 18,600/μL and C-reactive protein (CRP) level of 42 mg/L, raising suspicion of systemic infection. The Laboratory Risk Indicator for Necrotizing Fasciitis Score System was 6 points, indicating an intermediate risk for necrotizing soft-tissue infection.^[5]^ The patient showed general weakness and myalgia and was promptly admitted for both surgical and medical management of the lesion. Empirical treatment with intravenous cefazolin 1 g every 12 hours was initiated. Despite the patient’s previously unknown medical history, elevated blood glucose levels of 794 mg/dL and a hemoglobin A1C (HbA1C) of 15% indicated uncontrolled diabetes mellitus, which is the most common predisposing factor for necrotizing fasciitis.^[5]^ This necessitated insulin therapy alongside wound management.

**Figure 1.**
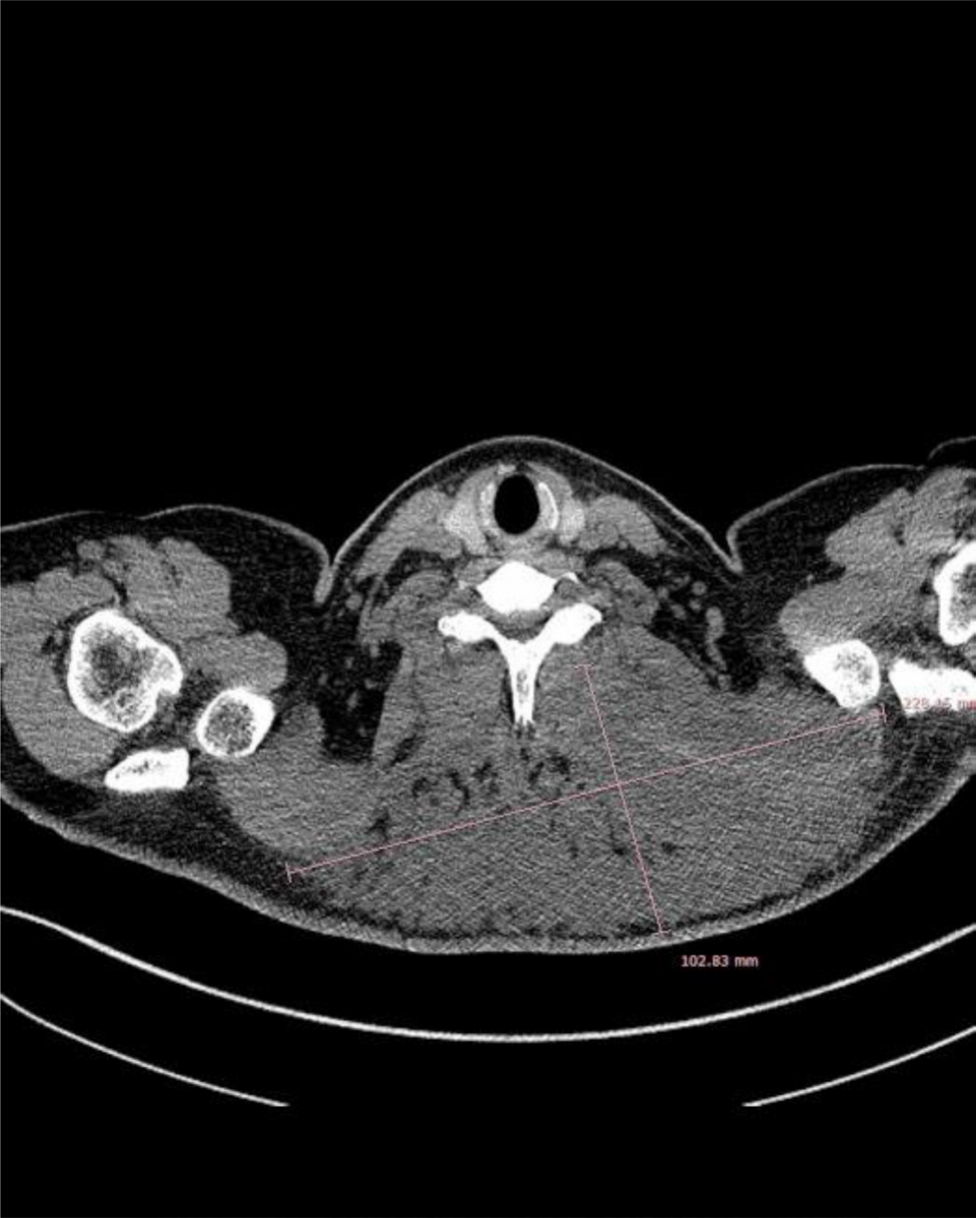
Initial chest computed tomography scan. The initial chest computed tomography scan of the patient’s back reveals a lesion measuring approximately 10.2 × 22.8 cm. A large cystic lesion surrounded by extensive soft-tissue infiltration is observed in the upper back. Additionally, the examination indicates involvement of the back muscle by the lesion.

**Figure 2.**
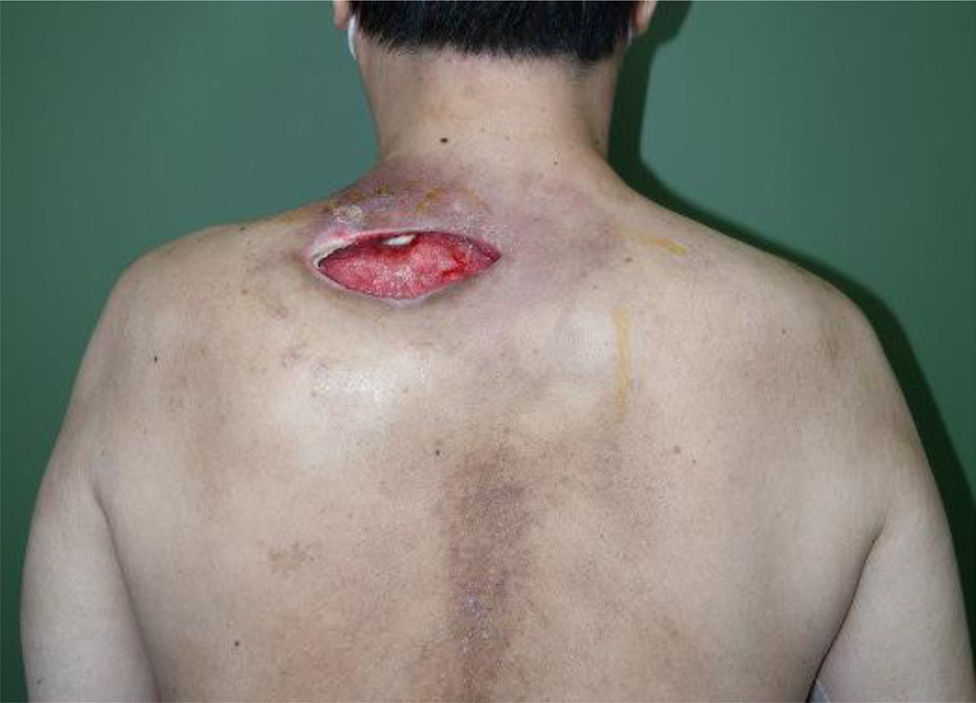
Postoperative gross photograph (incision and drainage). Postoperative (incision and drainage) gross photographs show the external wound to be approximately 10 × 6 cm, whereas the undermining surface measures approximately 18 × 12 cm.

**Figure 3.**
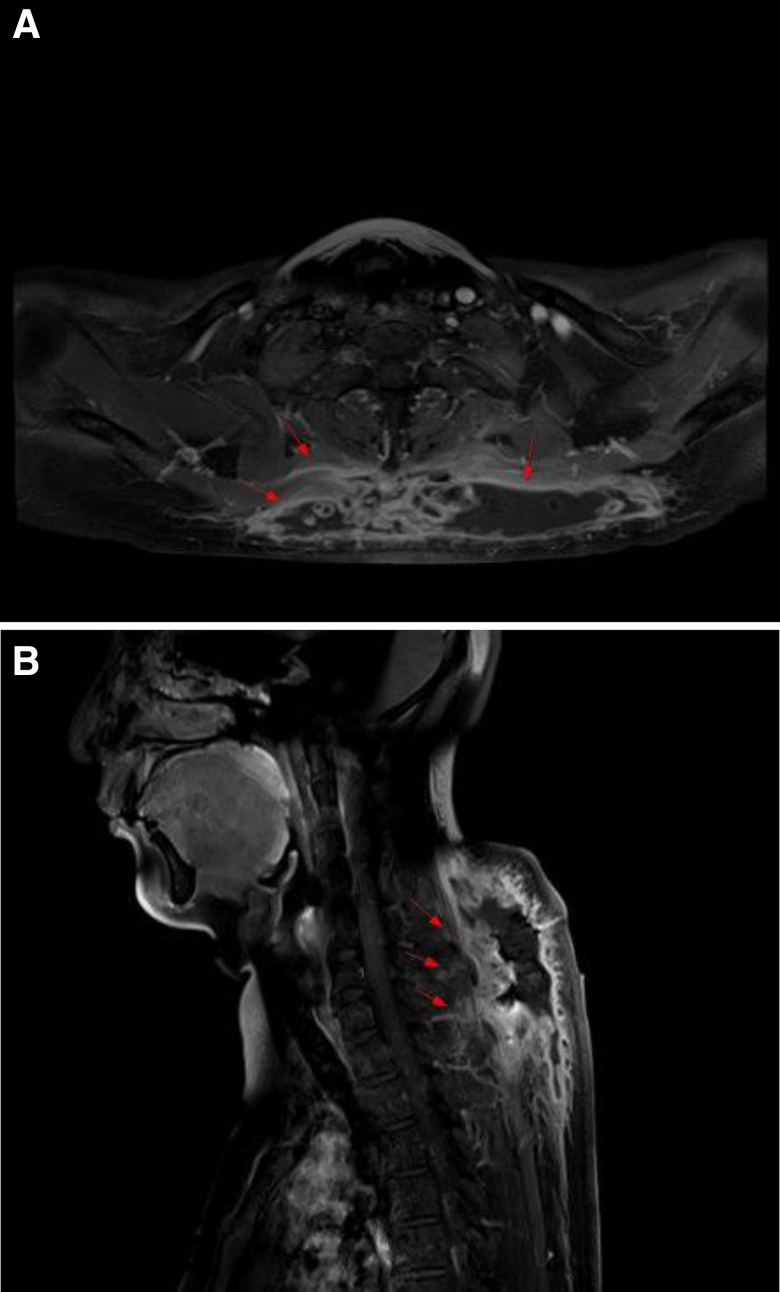
MRI following incision and drainage (upper back). MRI scan of the patient’s upper back after incision and drainage. (A) Skin and soft-tissue defect measuring 20 × 12 × 3.5 cm with necrotizing fasciitis of the left trapezius and levator scapulae. (B) Sagittal view showing superficial and deep fasciitis with involvement of the back muscles. MRI = magnetic resonance imaging.

To assess the extent and depth of the lesion, an emergency chest computed tomography scan was performed, revealing a lesion measuring approximately 10.2 × 22.8 cm with suspected involvement of the back muscles (Fig. 1). Incision and drainage were performed, and subsequent pus culture revealed the presence of *Staphylococcus aureus*. Consequently, concurrent treatment with intravenous cefazolin (1 g every 12 hours) was initiated. During the procedure, a substantial amount of pus was drained and the abscess was removed, exposing necrotic tissues. Despite the intervention, the wound failed to heal and continuous pus drainage persisted. Although the external wound appeared to be approximately 10 × 6 cm, further physical examination revealed an undermining surface measuring approximately 18 × 12 cm (Fig. 2) Subsequent enhanced magnetic resonance imaging revealed a large skin and soft-tissue defect measuring 20 × 12 × 3.5 cm, with involvement of the left trapezius and levator scapulae, along with evidence of superficial and deep fasciitis and pyomyositis, accompanied by abscess formation at the upper back. These findings confirmed the diagnosis of necrotizing fasciitis (Fig. 3A and B).

**Figure 4.**
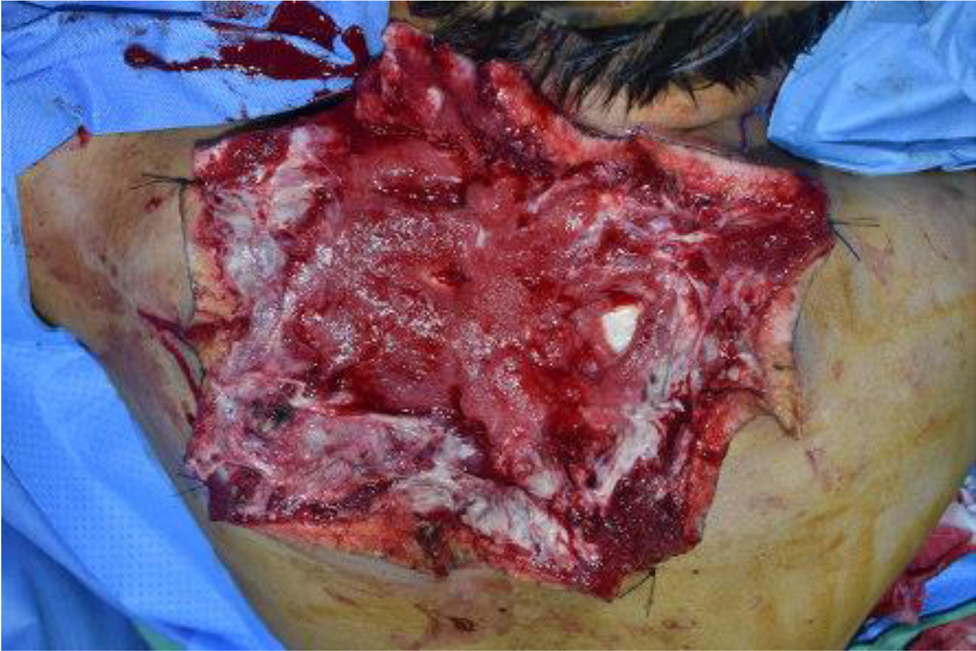
Intraoperative gross photography (debridement). Intraoperative gross photograph of the upper back. Five incisions were made to expose the undermined surface and necrotic tissues.

Two weeks later, extensive debridement was performed to completely excise the necrotic tissue. During this procedure, 5 incisions were made in a clockwise direction at the 1, 4, 6, 8, and 11 o’clock positions (Fig. 4). The necrotic tissue was removed to this extent, thereby completely exposing the undermining surface. This created a suitable wound for the application of NPWT. Intraoperative findings revealed necrotic tissue extending to the trapezius and levator scapulae muscles, with a significantly larger undermining surface than that apparent externally. Debridement of the necrotic tissue and fascia with bleeding control was concurrently performed for deep and extensive wounds. Although necrotizing fasciitis was evident, it had not extended into the bones. Prolonged courses of intravenous antibiotics were deemed necessary in consultation with the infectious disease department. Consequently, both surgical intervention and postoperative intravenous cefazolin were administered continuously for 4 weeks to alleviate the persistent infection.

Following debridement on the second postoperative day (POD#2), the CRP level was measured at 111.51 mg/L. However, on POD#6, it decreased to 42.81 mg/L. Similarly, the WBC count exhibited a decreasing trend from 9700/μL to 8700/μL. This sequential decline in inflammatory markers suggested an improvement in the patient’s systemic inflammation. Indeed, the patient experienced significant improvement in symptoms, such as fever and general weakness, consistent with the observed improvement in laboratory parameters.

The wound bed became clear after debridement. However, biofilm formation was observed on POD#1. Povidone-iodine-soaked gauze dressings were applied twice daily for a week. Consequently, the wound bed transitioned to a pinkish color, indicating favorable conditions for further surgical or nonsurgical approaches. The final undermined surface measured 35 × 30 cm, with a depth of 4 cm. The extensively undermined surface, measuring 35 × 30 × 4 cm, poses a significant challenge in determining the optimal approach for restoration and healing. We decided to focus on promoting granulation tissue growth to facilitate wound healing. This strategy was chosen to align with the patient’s preferences and minimize the risk of postoperative functional limitations. The subsequent course of treatment was aimed at encouraging natural tissue repair mechanisms.

Therefore, NPWT was initiated on POD#7, changed 3 times a week, and administered for 5 weeks. The CRP level showed a further decline to 13.01 mg/L on POD#9, eventually reaching 4.20 mg/L on POD#13 in a sequential pattern. The WBC count also declined from 7700/μL to 6200/μL. In consultation with an endocrinology team, insulin therapy was initiated for blood glucose control. Throughout hospitalization, blood sugar levels were stably maintained between 100 mg/dL and 130 mg/dL. Five days after discharge, the follow-up HbA1C level reduced to 5.9%, indicating effective glycemic control.

**Figure 5.**
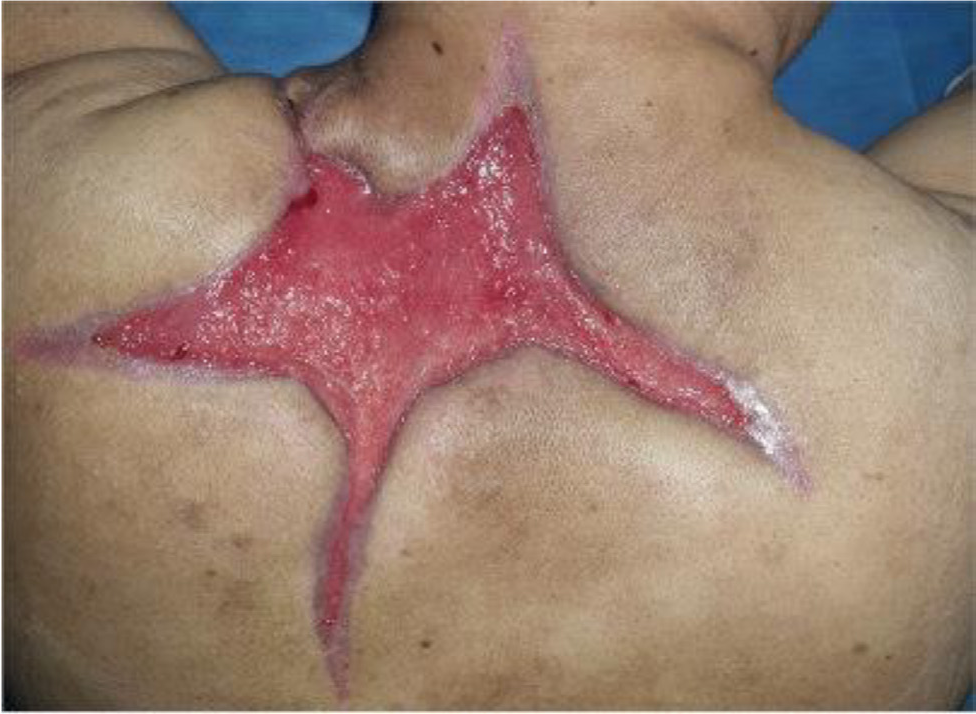
Preoperative gross photography after NPWT. Gross photographs after NPWT show the presence of granulation tissue as well as alignment with the surrounding skin and wound level. NPWT = negative pressure wound therapy.

**Figure 6.**
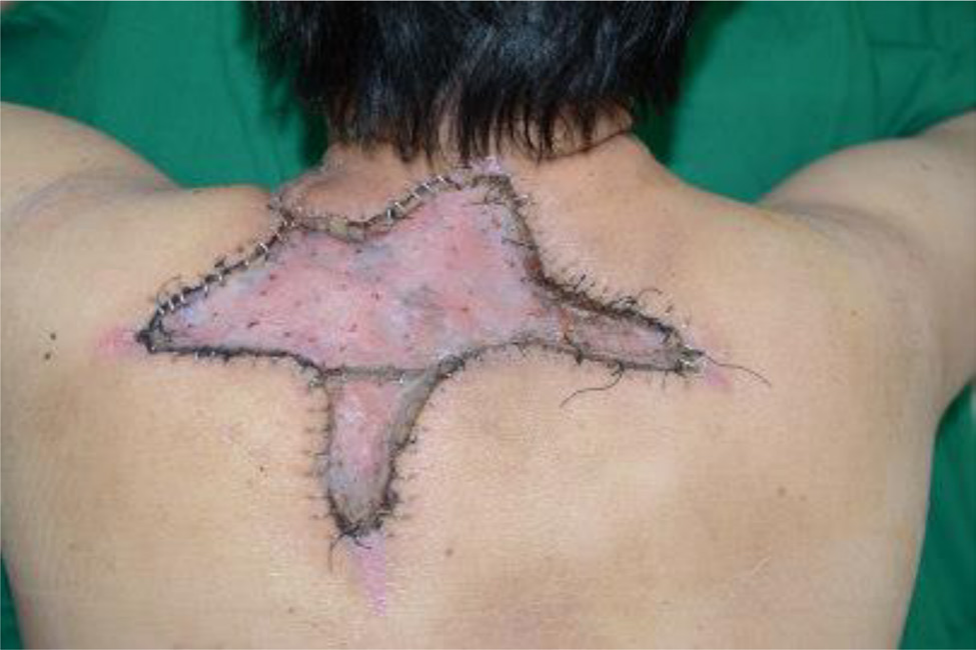
Gross photography 6 d after coverage with STSG. Postoperative gross photography 6 d after coverage with STSG shows graft take. STSG = split-thickness skin grafting.

**Figure 7.**
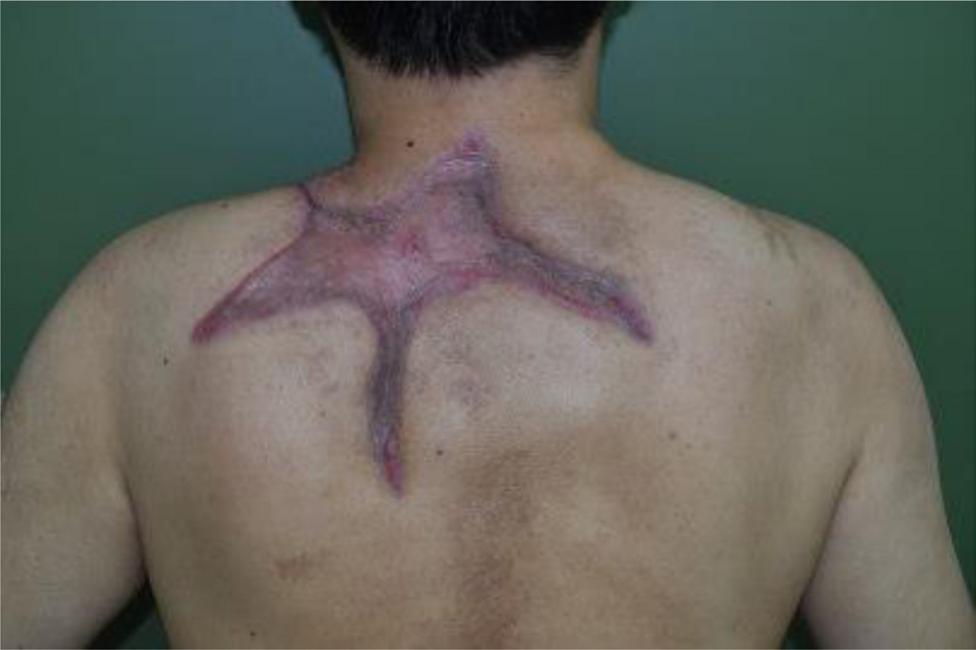
Gross photography 6 wk after coverage with STSG. Postoperative gross photography after coverage with STSG shows a healed wound without complications. STSG = split-thickness skin grafting.

The undermined surface decreased, granulation tissue formed, and after 4 weeks of NPWT, the surrounding skin and wound levels were nearly consistent with one another (Fig. 5) With no remaining dead space, a successful STSGing procedure was performed, covering an area of approximately 12 × 15 cm (Fig. 6) A skin graft was harvested from the right posterior thigh. The patient recovered without complications and was discharged 8 days after the surgery. At the 6-week post-surgery follow-up at the outpatient clinic, the patient exhibited a completely healed wound (Fig. 7).

## 3. Discussion

Epidermal cysts usually manifest as slow-growing, painless nodules beneath the skin. They typically arise in areas with a high concentration of sebaceous glands, such as the face, neck, back, and genitalia, usually not exceeding 5 cm.^[1]^ However, they can present with various clinical changes, including the development of extensive and deep lesions.^[4]^ The variability in clinical presentation further emphasizes the need to avoid overlooking these lesions and underscores the importance of employing appropriate clinical interventions tailored to the specific circumstances.

If left untreated, epidermal cysts can lead to several complications with potentially severe consequences. Neglected epidermal cysts can become inflamed, resulting in pain, redness, and swelling. Approaches to managing inflamed epidermal cysts include both conservative and procedural interventions.^[3]^ In instances where conservative measures prove inadequate or when recurrent inflammation occurs, procedural interventions such as incision and drainage or complete excision may become inevitable.

Furthermore, cyst rupture can lead to abscess formation and subsequent release of inflammatory contents into the surrounding tissues. This, in turn, may trigger an intense inflammatory response, potentially progressing to conditions such as cellulitis or necrotizing fasciitis in rare cases. The condition often arises from polymicrobial or monomicrobial infections, with *Staphylococcus aureus* being the second common culprit.^[6]^ The bacteria invade the fascial planes, releasing toxins that lead to widespread tissue destruction, compromised blood supply, and subsequent necrosis. Failure to promptly treat necrotizing fasciitis can result in fatalities. Early diagnosis is challenging, and the lesion is often mistaken for a simple abscess. Because patients with comorbidities, such as diabetes mellitus, renal failure, and liver cirrhosis, are affected by this condition, concurrent medical management should also be considered. In such cases, immediate extensive debridement is essential and has an impact on the patient’s survival rate.^[7]^

In this case, the patient’s decision to leave the epidermal cyst untreated resulted in its rapid enlargement and eventual rupture, leading to symptoms of systemic infection upon presentation. Notably, the reported cyst was larger than typical epidermal cysts, with considerable depth, invading the underlying muscles. The presence of necrotizing fasciitis and the formation of a significantly extensive defect made this case uncommon. The patient’s neglect of the epidermal cyst resulted in a complex and severe presentation.

Although the emergence of a substantial wound due to the epidermal cyst was noteworthy, it posed considerable challenge in terms of wound coverage. In this case, the decision was made to fill the defect by employing NPWT followed by STSG. Ultimately, both the patient and the medical team were satisfied with the results.

The primary objective of this case report was to highlight the development of an unusual wound resulting from an epidermal cyst. Although typical complications such as rupture or infection may occur, cases in which these complications result in extensive skin defects and necrotizing fasciitis are rare. In such cases, prompt and appropriate interventions, including debridement and wound coverage, are crucial for successful treatment.

While we have presented the patient’s progress during a 6-week follow-up period, the assessment of potential long-term complications and recurrence remains limited. Future research should consider conducting long-term studies involving similar patients to better understand the extended outcomes and potential late complications associated with this condition.

The rarity of this presentation emphasizes the importance of recognizing and addressing the complications associated with epidermal cysts, particularly when they lead to severe consequences, such as substantial skin defects. This case highlights the need for a comprehensive and tailored approach to manage unusual wounds arising from benign skin lesions to ensure optimal outcomes and patient satisfaction.

## Author contributions

**Conceptualization:** Jae Hyun Kim, Jun Il Kim.

**Formal analysis:** Jae Hyun Kim, Jun Il Kim.

**Writing – original draft:** Jae Hyun Kim, Jun Il Kim.

**Writing – review & editing:** Chan Min Chung.
